# Efficacy and Safety of Immuno-Oncology Plus Tyrosine Kinase Inhibitors as Late-Line Combination Therapy for Patients with Advanced Renal Cell Carcinoma

**DOI:** 10.3390/jcm13123365

**Published:** 2024-06-07

**Authors:** Shuzo Hamamoto, Yoshihiko Tasaki, Toshiharu Morikawa, Taku Naiki, Toshiki Etani, Kazumi Taguchi, Shoichiro Iwatsuki, Rei Unno, Tomoki Takeda, Takashi Nagai, Kengo Kawase, Yoshihisa Mimura, Yosuke Sugiyama, Atsushi Okada, Yoko Furukawa-Hibi, Takahiro Yasui

**Affiliations:** 1 Department of Nephro-Urology, Nagoya City University Graduate School of Medical Sciences, 1 Kawasumi, Mizuho-cho, Mizuho-ku, Nagoya 467-8601, Japan; t-mrkw@med.nagoya-cu.ac.jp (T.M.); naiki@med.nagoya-cu.ac.jp (T.N.); uroetani@med.nagoya-cu.ac.jp (T.E.); ktaguchi@med.nagoya-cu.ac.jp (K.T.); s001013i@yahoo.co.jp (S.I.); unno@med.nagoya-cu.ac.jp (R.U.); tkd7888@med.nagoya-cu.ac.jp (T.T.); tkshng73@med.nagoya-cu.ac.jp (T.N.); kawase@med.nagoya-cu.ac.jp (K.K.); a-okada@med.nagoya-cu.ac.jp (A.O.); yasui@med.nagoya-cu.ac.jp (T.Y.); 2 Department of Clinical Pharmaceutics, Nagoya City University Graduate School of Medical Sciences, 1 Kawasumi, Mizuho-cho, Mizuho-ku, Nagoya 467-8601, Japan; phtasaki@med.nagoya-cu.ac.jp (Y.T.); phmimura@med.nagoya-cu.ac.jp (Y.M.); phsugi@med.nagoya-cu.ac.jp (Y.S.); yokoh@med.nagoya-cu.ac.jp (Y.F.-H.)

**Keywords:** immuno-oncology, renal cell carcinoma, tyrosine kinase inhibitor

## Abstract

**Background/Objectives:** Immuno-oncology plus tyrosine kinase inhibitor (IO+TKI) combination therapy is an essential first-line therapy for advanced renal cell carcinoma (RCC). However, reports of its efficacy and safety as late-line therapy are lacking. This study aimed to examine the efficacy and safety of IO+TKI combination therapy as a late-line therapy for patients with RCC. **Methods:** We retrospectively examined 17 patients with RCC who received IO+TKI combination therapy as a second-line therapy or beyond (pembrolizumab plus axitinib, *n* = 10; avelumab plus axitinib, *n* = 5; nivolumab plus cabozantinib, *n* = 2). **Results:** The overall response and disease control rates of IO+TKI combination therapy were 29.4% and 64.7%, respectively. The median overall survival was not attained. Progression-free survival was 552 days, and 94.1% of patients (*n* = 16) experienced adverse effects (AEs) of any grade; moreover, 41.2% of patients (*n* = 7) experienced grade ≥ 3 immuno-related AEs. **Conclusions:** IO+TKI combination therapy may be a late-line therapy option for RCC.

## 1. Introduction

Advanced renal cell carcinoma (RCC) is a rare and refractory cancer. Large-scale cancer statistics, which estimate new cancer cases and deaths in the United States, showed that approximately 4% (83,190 of 2,001,140 cases) of all cancer cases diagnosed each year were RCC [1]. The estimated 5-year survival rates for RCC between 2014 and 2019 were 93% for localized cases, 74% for regional cases, and 17% for distant cases [1]. According to the National Cancer Center Japan, approximately 3% (30,458 of 999,075 cases) of all cancer cases diagnosed each year in Japan were RCC. Additionally, the estimated 5-year survival rates for RCC in Japan were 94.3% for localized cases, 53.6% for regional cases, and 12.4% for distant cases. These statistical data indicate that RCC is a rare cancer that is associated with a poor prognosis. One reason for its poor prognosis is the lack of efficient therapy for RCC. However, recently, immuno-oncology (IO) therapy was approved for RCC, thus dramatically changing the treatment strategy and prognosis of such patients [2,3,4,5,6,7,8].

The European Association of Urology and the National Comprehensive Cancer Network (NCCN) guidelines strongly recommend IO plus tyrosine kinase inhibitor (IO+TKI) combination therapy or IO combination therapy as the standard first-line therapy for RCC [2,3,4,5,6,7,8]. Patients who cannot tolerate IO therapy receive TKI monotherapy as the first-line therapy for RCC [8]. There is no consensus regarding whether IO+TKI combination therapy or IO combination therapy is the optimal treatment for patients with RCC. Based on the International Metastatic Renal Cell Carcinoma Database Consortium (IMDC) risk category, histological subtype, and patient condition, physicians can use several treatments as first-line therapy for RCC. Patients with disease progression after IO+TKI combination therapy or IO combination therapy as the standard first-line therapy for RCC require a new treatment modality as late-line therapy. However, although physicians can choose from many treatments as first-line therapy, the available late-line therapies may not be sufficiently effective.

IO+TKI combination therapy is a rational immunological treatment option. TKI can impose anti-tumor effects alone and also support the effects of IO through the suppression of regulatory T cells [9,10]. Large-scale clinical trials have shown that IO+TKI combination therapy is associated with better clinical outcomes [2,3,4,5,7,8]. For instance, the median progression-free survival (mPFS) of nivolumab plus cabozantinib is significantly longer than that of sunitinib (16.6 months vs. 8.3 months) [2]. Compared with sunitinib, pembrolizumab plus axitinib significantly decreases the risk of disease progression or death (hazard ratio: 0.69) [4]. Therefore, IO+TKI combination therapies, such as cabozantinib plus nivolumab, pembrolizumab plus lenvatinib, pembrolizumab plus axitinib, and avelmab plus axitinib, were approved and used as first-line therapy for RCC in Japan in 2019 [2,3,4,5]. Additionally, the NCCN guidelines describe the use of IO+TKI combination therapy after late-line therapy, regardless of whether the patient has received IO therapy [7]. However, the NCCN guidelines do not strongly recommend IO+TKI combination therapy after late-line therapy. One reason for this is the lack of reports on the efficacy and safety of IO+TKI combination therapy as late-line therapy. Therefore, this study aimed to investigate the efficacy and safety of IO+TKI combination therapy as late-line therapy for patients with advanced RCC.

## 2. Materials and Methods

### 2.1. Patients and Treatment

Seventeen patients were retrospectively analyzed at Nagoya City University Hospital between April 2020 and September 2023. We enrolled patients with RCC who received IO+TKI combination therapy as late-line therapy after one or more regimens of TKI monotherapy (sunitinib, axitinib, pazopanib, and sorafenib), nivolumab monotherapy, ipilimumab plus nivolumab, and mammalian target of rapamycin (mTOR) inhibitors (temsirolimus and everolimus). IO+TKI combination therapy included pembrolizumab (200 or 400 mg/kg every 3 or 6 weeks) plus axitinib (10 mg twice daily), avelumab (10 mg/kg every 2 weeks) plus axitinib (10 mg twice daily), and nivolumab (240 or 480 mg/kg every 2 or 4 weeks) plus cabozantinib (40 mg once daily). Physicians selected the therapy based on the patients’ conditions. Patients who received IO+TKI combination therapy as first-line therapy were excluded. Experienced pathologists diagnosed RCC based on the results of histological examinations. Two cases involving unknown histological subtypes were diagnosed as RCC by an experienced radiologist and urologist who used computed tomography and magnetic resonance imaging to evaluate the patients. All patients were followed-up until death or loss of contact. Overall survival (OS) was analyzed as the period from the start of IO+TKI combination therapy until death or the last follow-up. The treatment response was assessed using the Response Evaluation Criteria in Solid Tumors (RECIST) version 1.1 as a complete response (CR), partial response (PR), stable disease (SD), or progressive disease (PD) [11]. We examined the adverse effects (AEs) induced by IO or TKI using blood sample tests and clinical assessments. The AEs were graded according to the National Cancer Institute Common Terminology Criteria for Adverse Events version 5.0.

### 2.2. Statistical Analysis

*p* < 0.05 was considered statistically significant. The Kaplan–Meier method and log-rank tests were performed to analyze the median OS and mPFS. Statistical analyses were performed using GraphPad Prism 9 software.

## 3. Results

### 3.1. Patient Characteristics

Patient characteristics before IO+TKI combination therapy are shown in Table 1. The characteristics of each patient before IO+TKI combination therapy are shown in Table 2. Seventeen patients with RCC were enrolled in this study. The median follow-up duration was 515 days (range, 75–1216 days). The median period from the diagnosis of RCC to the start of IO+TKI combination therapy was 1687 days (range, 127–5655 days). The median patient age was 73 years (range, 55–88 years). The proportions of men and women were 70.6% (*n* = 12) and 29.4% (*n* = 5), respectively. Comorbidities and disease histories before IO+TKI combination therapy were hypertension (29.4%; *n* = 5), diabetes mellitus (17.6%; *n* = 3), arrhythmia (11.8%; *n* = 2), dyslipidemia (11.8%; *n* = 2), angina (5.9%; *n* = 1), cataracts (5.9%; *n* = 1), chronic heart failure (5.9%; *n* = 1), gastric ulcer (5.9%; *n* = 1), glaucoma (5.9%; *n* = 1), Guillain-Barre syndrome (5.9%; *n* = 1), hemorrhoids (5.9%; *n* = 1), hyperuricemia (5.9%; *n* = 1), myocardial infarction (5.9%; *n* = 1), rheumatoid arthritis (5.9%; *n* = 1), and uterine fibroids (5.9%; *n* = 1). Additionally, 35.3% (*n* = 6) of patients did not have comorbidities or disease histories before IO+TKI combination therapy. All patients had stage IV disease. The patients were categorized as favorable risk (23.5%; *n* = 4), intermediate risk (58.8%; *n* = 10), and poor risk (11.8%; *n* = 2) according to the IMDC risk categories. Among the histological subtypes, the highest proportion of patients had the clear cell type (82.3%; *n* = 14), followed by the non-clear cell type (5.9%; *n* = 1). The histological subtype of 11.8% (*n* = 2) of patients was unknown. None of the patients exhibited sarcomatoid changes. The metastatic sites were the liver (23.5%; *n* = 4), lungs (76.5%; *n* = 13), and others such as the bones, brain, adrenal glands, pancreas, prostate, pleura, retroperitoneum, ileocecum, peritoneum, and parathyroid (70.6%; *n* = 12).

### 3.2. Therapeutic Features

The therapeutic features of the patients are summarized in Table 3. The administered treatments were pembrolizumab + axitinib (58.8%; *n* = 10), avelumab + axitinib (29.4%; *n* = 5), and nivolumab + cabozantinib (11.8%; *n* = 2). The median numbers of courses of these therapies were 15.5 (range, 3–51) for pembrolizumab + axitinib, 23 (range, 6–38) for avelumab + axitinib, and 5.5 (range, 2–9) for nivolumab + cabozantinib. The median daily dosages of TKI were 5.5 mg (range, 2.5–7.3 mg) for Axitinib and 35 mg (range, 30–40 mg) for Cabozantinib. Among those who received IO+TKI combination therapy, the highest proportion received it as second-line therapy (47.0%; *n* = 8), followed by third-line therapy (17.6%; *n* = 3), fourth-line therapy (5.9%; *n* = 1), fifth-line therapy (5.9%; *n* = 1), and sixth-line therapy and beyond (23.5%; *n* = 4). The therapies administered before IO+TKI combination therapy were nivolumab monotherapy (52.9%; *n* = 9 cases), sunitinib (52.9%; *n* = 9 cases), ipilimumab plus nivolumab (41.2%; *n* = 7 cases), axitinib (35.3%; *n* = 6 cases), everolimus (29.4%; *n* = 5 cases), Pazopanib (17.6%; *n* = 3 cases), sorafenib (17.6%; *n* = 3 cases), and temsirolimus (5.9%; *n* = 1 case). The subsequent therapies administered after IO+TKI combination therapy included axitinib (17.6%; *n* = 3), cabozantinib (5.9%; *n* = 1), and nivolumab monotherapy (5.9%; *n* = 1). 

The therapeutic features of each patient are described in Table 4. Among eight patients who received IO+TKI combination therapy as second-line therapy, seven received ipilimumab plus nivolumab and one received pazopanib as first-line therapy before IO+TKI combination therapy (cases 1, 4, 12, 13, 14, 15, 16, and 17). The three patients who received IO+TKI combination therapy as third-line therapy received TKI monotherapy (sunitinib and pazopanib) and nivolumab monotherapy before IO+TKI combination therapy (cases 3, 7, and 8). One patient who received IO+TKI combination therapy as fourth-line therapy received TKI monotherapy (sunitinib and pazopanib) and nivolumab monotherapy before IO+TKI combination therapy (case 9). The five patients who received IO+TKI combination therapy as fifth-line therapy and beyond received two or more types of TKI monotherapy (sunitinib, axitinib, sorafenib, and/or pazopanib), nivolumab monotherapy, and mTOR inhibitors before IO+TKI combination therapy (cases 2, 5, 6, 10, and 11).

### 3.3. Efficacy and Safety of IO+TKI Combination Therapy

**Figure 1:**
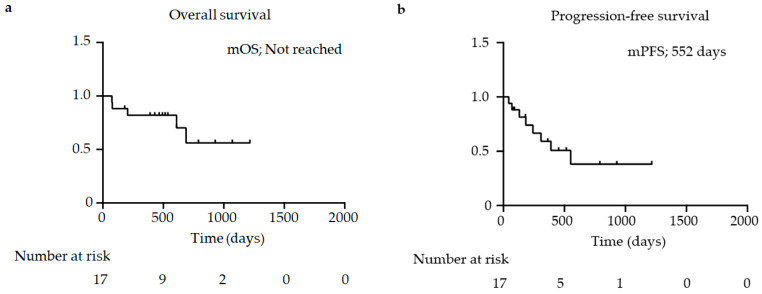
Kaplan–Meier survival curves for (**a**) overall survival (*n* = 17) and (**b**) progression-free survival (*n* = 17). (**a**,**b**) Log-rank test. mOS, median overall survival; mPFS, median progression-free survival.

We analyzed the efficacy of IO+TKI combination therapy and found the following response rates: CR, 0.0% (*n* = 0); PR, 29.4% (*n* = 5); SD, 35.3% (*n* = 6); and PD, 35.3% (*n* = 6). The proportions of objective response rates and disease control rates were 29.4% (*n* = 5) and 64.7% (*n* = 11), respectively (Table 5). The Kaplan–Meier method showed that the median OS was not reached and that the mPFS was 552 days (Figure 1a,b). 

Table 6 presents the details of the AEs. Of the patients included in this study, 94.1% (*n* = 16) experienced AEs. Among these AEs, 41.2% (*n* = 7) were grade ≥3. The incidences of AEs of any grade were as follows: diarrhea, 70.6% (*n* = 12); hypertension, 23.5% (*n* = 4); fever, 17.6% (*n* = 3); hoarseness, 17.6% (*n* = 3); decreased appetite, 11.8% (*n* = 2); rash, 11.8% (*n* = 2); fatigue, 11.8% (*n* = 2); pneumonitis, 11.8% (*n* = 2); and other disorders (nausea, alopecia areata, hand–foot skin reaction, diabetes mellitus, thyroid dysfunction, edema, serum creatine elevation, hyponatremia, myasthenia gravis, and proteinuria), 5.9% (*n* = 1). Grade ≥3 AEs were diarrhea, fatigue, pneumonitis, hand–foot skin reaction, diabetes mellitus, edema, hyponatremia, myasthenia gravis, and proteinuria (5.9%; *n* = 1).

The details of the AEs of each patient are shown in Table 7. Ten patients experienced three or more AEs (cases 1, 2, 4, 5, 7, 11, 12, 14, 15, and 16). Three patients experienced two AEs (cases 3, 13, and 17) and three patients experienced one AE (cases 8, 9, and 10). One patient did not experience any AEs (case 6). No patients experienced worsened comorbidities or had disease histories attributable to IO+TKI combination therapy.

The reasons for discontinuing treatment included disease progression (47.0%; *n* = 8) and AEs (17.6%; *n* = 3) (Table 8). Furthermore, 35.3% (*n* = 6) of patients enrolled in the current study experienced AEs attributable to IO therapy before IO+TKI combination therapy. Among these patients, 11.8% (*n* = 2) experienced further severe AEs after IO+TKI combination therapy and discontinued therapy. 

### 3.4. Analysis of the Association between Efficacy and the IMDC Risk

**Figure 2:**
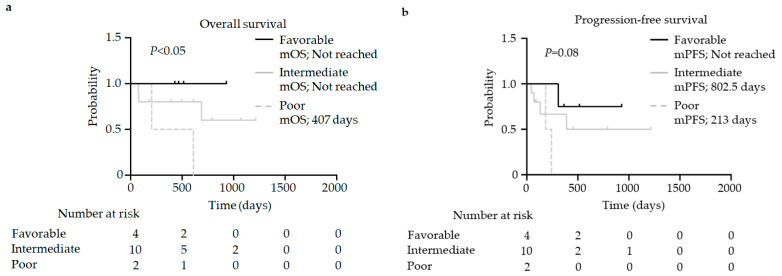
Kaplan–Meier survival curves for (**a**) overall survival (favorable IMDC risk group: *n* = 4; intermediate IMDC risk group: *n* = 10; poor IMDC risk group: *n* = 2) and (**b**) progression-free survival (favorable IMDC risk group: *n* = 4; intermediate IMDC risk group: *n* = 10; poor IMDC risk group: *n* = 2) of patients with renal cell carcinoma. (**a**,**b**) Log-rank test. IMDC, International Metastatic Renal Cell Carcinoma Database Consortium; mOS: median overall survival; mPFS: median progression-free survival.

Finally, we examined the association between the survival time and IMDC risk. The median OS and mPFS of patients with a poor IMDC risk were associated with poor clinical survival compared to those of patients with favorable and intermediate IMDC risks (OS: not reached vs. not reached vs. 407 days, *p* < 0.05; PFS: not reached vs. 802.5 days vs. 213 days, *p* = 0.08) (Figure 2a,b). 

## 4. Discussion

We investigated the efficacy and safety of IO+TKI combination therapy as a late-line therapy. To date, IO+TKI combination therapy has been the first-line therapy [2,3,4,5]. A large-scale clinical trial reported mPFS from 13.8 to 23.9 months [2,3,4,5]. The current study demonstrated that the efficacy of IO+TKI combination therapy as a late-line therapy may be similar to its efficacy as a first-line therapy. Among TKI monotherapies, cabozantinib and axitinib are mainly selected as a late-line therapy for RCC. During a phase 3 trial, the mPFS of patients who received cabozantinib and axitinib were 7.4 months and 6.7 months, respectively [12,13]. However, these patients did not receive IO+TKI combination therapy or IO combination therapy as a previous systemic therapy, which may differ from the current treatment strategy. Several studies have reported real-world data regarding cabozantinib or axitinib as a late-line therapy [14,15,16,17,18,19]. Suzuki et al. reported that the mPFS of patients who received axitinib as a second-line therapy was 10.3 months [14]. Bodnar et al. and Stukalin et al. reported that the mPFS of patients who received cabozantinib as a second-line therapy were 12.5 months and 7.39 months, respectively [15,16]. Furthermore, Gan et al. and Domański et al. examined the efficacy of cabozantinib as a second-line therapy [17,18]. According to Gan et al., the mPFS associated with second-line therapy was 7.3 months, that associated with third-line therapy was 7.0 months, and that associated with fourth-line therapy was 8.0 months [17]. Domański et al. reported that the mPFS associated with second-line therapy and beyond was 11 months [18]. Procopio et al. evaluated the efficacy of cabozantinib for patients who received IO+TKI combination therapy or IO combination therapy as previous systemic therapy and reported an mPFS of 8.3 months [19]. The current study showed that the efficacy of IO+TKI combination therapy as late-line therapy may be comparable to or better than that of cabozantinib or axitinib therapy. Considering these results regarding the efficacy of first-line and second-line therapies and beyond, our study suggested that IO+TKI combination therapy may be suitable as a late-line therapy.

The safety of IO+TKI combination therapy as a first-line therapy has been reported in phase 3 trials [2,3,4,5]. The incidences of any grade and grade ≥3 AEs were 99.7% and 75.3% for nivolumab + cabozantinib, 99.7% and 82.4% for lenvatinib + pembrolizumab, 98.4% and 75.8% for pembrolizumab + axitinib, and 99.5% and 71.2% for avelumab + axitinib, respectively. Similar to these phase 3 trials, our study found that diarrhea had the highest incidence among AEs of any grade. Other AEs with high incidences were hypertension, decreased appetite, and fatigue. The current study showed that the incidences of any grade and grade ≥3 AEs were 94.1% and 41.2%, respectively. Additionally, diarrhea had the highest incidence in phase 3 trials. Phase 3 trials also reported the following incidences of treatment discontinuation attributable to AEs [2,3,4,5]: 19.7% for nivolumab + cabozantinib therapy; 37.2% for lenvatinib + pembrolizumab; 30.5% for pembrolizumab + axitinib; and 7.6% for avelumab + axitinib [2,3,4,5]. Furthermore, 17.6% of patients discontinued treatment because of AEs. In summary, the incidences of treatment discontinuation and AEs and the AE profiles observed during our study were similar to those observed during these clinical trials. Interestingly, 35.3% (*n* = 6) of patients who received IO therapy before IO+TKI combination therapy experienced immune-related AEs as a result of prior therapy. Nevertheless, it was difficult to continue treatment for 11.8% of patients because of further severe immune-related AEs attributable to IO+TKI combination therapy. Although decisions regarding the treatment of patients with comorbidities, disease histories, and prior therapy-related AEs require caution, the current study suggested that IO+TKI combination therapy as a late-line therapy is safe and tolerable.

Biomarkers that predict the efficacy of IO+TKI combination therapy have been reported [20,21,22,23,24,25]. For example, the expression levels of cyclin-dependent kinases 5 and 6, which play important roles in the cell cycle, are associated with efficacy and tumor-infiltrating immune cells [20,23]. Wang et al. showed that elevated RUNX3 expression levels in tumors are associated with poor efficacy [25]. However, those studies focused on the examination of tumors, and no reports have focused on blood parameters that can be easily investigated. Furthermore, no biomarkers are available for clinical use. Previously, we investigated biomarkers that could predict the effectiveness of IO therapy [26,27,28]. In the current study, the IMDC risk may have been associated with efficacy, as in our previous study [26]. These findings may predict efficacy and safety and should be considered when determining treatment strategies for patients with RCC.

There have been only two studies of the efficacy and safety of IO+TKI combination therapy as a late-line therapy [29,30]. However, those studies retrospectively examined a small number of patients. Dizman et al. examined the efficacy and safety of IO+TKI combination therapy for 38 patients who received pembrolizumab + aitinib treatment [29] and revealed that the mPFS was 9.7 months and that any grade, grade 3, and grade 4 AEs occurred in 86.8, 18.4%, and 6.4% patients, respectively. Yang et al. reported that the mPFS of second-line, third-line, and fourth-line therapies and beyond were 14.2 months, 10.1 months, and 6.8 months, respectively, and that the incidence of grade ≥3 AEs was 52.0% [30]. The efficacy and safety observed during the current study were comparable to those reported by previous studies. Therefore, the results of previous studies support our data that indicated that IO+TKI combination therapy may be suitable as a late-line therapy.

The present study had some limitations. First, it examined a small number of patients; therefore, a large-scale study is required to confirm the validity of our findings. Second, the retrospective design may have resulted in selection bias; therefore, prospective studies are warranted.

## 5. Conclusions

IO+TKI combination therapy may be used as a late-line therapy for patients with RCC who have received one or more other regimens, such as TKI monotherapy, nivolumab monotherapy, IO combination therapy, or mTOR inhibitors.

## Figures and Tables

**Table 1:** Patient characteristics.

Characteristics	
Total, *n* (%)	17 (100)
Age, median (range)	73 (55–88)
Sex, *n* (%)	
Male	12 (70.6)
Female	5 (29.4)
Comorbidities and disease histories	
Hypertension	5 (29.4)
Diabetes mellitus	2 (11.8)
Arrhythmia	2 (11.8)
Dyslipidemia	2 (11.8)
Angina	1 (5.9)
Cataracts	1 (5.9)
Chronic heart failure	1 (5.9)
Gastric ulcer	1 (5.9)
Glaucoma	1 (5.9)
Guillain–Barre syndrome	1 (5.9)
Hemorrhoids	1 (5.9)
Hyperuricemia	1 (5.9)
Myocardial infarction	1 (5.9)
Rheumatoid arthritis	1 (5.9)
Uterine fibroids	1 (5.9)
None	7 (41.2)
Stage	
I	0 (0.0)
II	0 (0.0)
III	0 (0.0)
IV	17 (100)
IMDC risk group, *n* (%)	
Favorable	4 (23.5)
Intermediate	10 (58.8)
Poor	2 (11.8)
Not evaluable	1 (5.9)
Histological subtype, *n* (%)	
Clear cell	14 (82.3)
Non-clear cell (Bellini duct carcinoma)	1 (5.9)
Unknown	2 (11.8)
Sarcomatoid change	
No	17 (100)
Yes	0 (0.0)
Metastasis site, liver, *n* (%)	
No	13 (76.5)
Yes	4 (23.5)
Metastasis site, lung, *n* (%)	
No	4 (23.5)
Yes	13 (76.5)
Metastasis site, others, *n* (%)	
No	5 (29.4)
Yes	12 (70.6)

IMDC: International Metastatic Renal Cell Carcinoma Database Consortium.

**Table 2:** Characteristics of each patient before IO+TKI combination therapy.

Case Number	Age	Sex	Comorbidities and Disease Histories	Period from the RCC Diagnosis to the Start of IO+TKI Combination Therapy (Days)	Stage	IMDC Risk Group	Histological Subtype	Metastasis Site
1	74	Female	Diabetes	177	IV	Favorable	Clear cell	Lung
			Dyslipidemia					
			Hypertension					
2	85	Male	Chronic heart failure	2401	IV	Favorable	Clear cell	Lung
			Hypertension					
3	88	Male	None	5193	IV	Intermediate	Clear cell	Lung
								Peritoneum
4	85	Female	None	258	IV	Intermediate	Unknown	Lung
5	67	Male	None	5655	IV	Intermediate	Clear cell	Liver
								Pancreas
								Ileocecum
								Retroperitoneum
6	76	Female	Hypertension	3712	IV	Intermediate	Clear cell	Lung
			Uterine fibroids					Bone
7	80	Male	Cataract	3986	IV	Favorable	Clear cell	Lung
			Gastric ulcer					Bone
			Glaucoma					Pancreas
			Hemorrhoids					Adrenal glands
8	61	Male	Diabetes	2647	IV	Favorable	Clear cell	Lung
			Dyslipidemia					Pleura
								Parathyroid
			Hyperuricemia					Prostate
9	73	Male	Hypertension	2043	IV	Intermediate	Clear cell	Lung
								Bone
			Rheumatoid arthritis					Adrenal glands
10	67	Female	Hypertension	3328	IV	Not evaluable	Unknown	Lung
								Liver
								Bone
11	65	Female	None	1687	IV	Intermediate	Clear cell	Bone
12	68	Male	Arrhythmia	920	IV	Intermediate	Clear cell	Adrenal glands
			Guillain-Barre syndrome					Bone
13	81	Male	Angina	1246	IV	Intermediate	Bellini duct carcinoma	Lung
								Liver
14	59	Male	None	385	IV	Intermediate	Clear cell	Bone
15	55	Male	None	168	IV	Intermediate	Clear cell	Lung
								Bone
16	79	Male	Arrhythmia	127	IV	Poor	Clear cell	Lung
			Myocardial infarction					Liver
17	58	Male	None	412	IV	Poor	Clear cell	Lung
								Bone
								Brain
								Adrenal glands

IMDC: International Metastatic Renal Cell Carcinoma Database Consortium.

**Table 3:** Therapeutic features of the included patients.

Characteristics	
Total, *n* (%)	17 (100)
IO+TKI combination therapy, *n* (%)	17 (100)
Pembrolizumab + axitinib	10 (58.8)
Avelumab + axitinib	5 (29.4)
Nivolumab + cabozantinib	2 (11.8)
Median of number of courses, (range)	
Pembrolizumab + axitinib	15.5 (3–51)
Avelumab + axitinib	23 (6–38)
Nivolumab + cabozantinib	5.5 (2–9)
Median daily dosage of TKI, mg (range)	
Axitinib	5.5 (2.2–7.3)
Cabozantinib	35 (30–40)
Line of IO+TKI combination therapy	17 (100)
Second line	8 (47.0)
Third line	3 (17.6)
Fourth line	1 (5.9)
Fifth line	1 (5.9)
Sixth line and beyond	4 (23.5)
Therapy before IO+TKI combination therapy	
Specifications, *n* (%)	
Nivolumab monotherapy	9 (52.9)
Sunitinib	9 (52.9)
Ipilimumab plus nivolumab	7 (41.2)
Axitinib	6 (35.3)
Everolimus	5 (29.4)
Pazopanib	3 (17.6)
Sorafenib	3 (17.6)
Temsirolimus	1 (5.9)
Subsequent therapy after IO+TKI combination therapy	
Specifications, *n* (%)	
Axitinib	3 (17.6)
Cabozantinib	1 (5.9)
Nivolumab monotherapy	1 (5.9)

IO+TKI: immuno-oncology plus tyrosine kinase inhibitor.

**Table 4:** Therapeutic features of each patient.

Case Number	First Line	Second Line	Third Line	Fourth Line	Fifth Line	Sixth Line	Seventh Line	Eighth Line
1	Ipilimumab + nivolumab	Pembrolizumab + axitinib	Cabozantinib	-	-	-	-	-
2	Sunitinib	Axitinib	Everolimus	Nivolumab monotherapy	Pembrolizumab + axitinib	Nivolumab monotherapy	-	-
3	Pazopanib	Nivolumab monotherapy	Avelumab + axitinib	Axitinib	-	-	-	-
4	Ipilimumab + nivolumab	Avelumab + axitinib	-	-	-	-	-	-
5	Sorafenib	Sunitinib	Everolimus	Axitinib	Nivolumab monotherapy	Pazopanib	Temsirolimus	Pembrolizumab + axitinib
6	Sunitinib	Everolimus	Sorafenib	Sunitinib	Nivolumab monotherapy	Pembrolizumab + axitinib	-	-
7	Sunitinib	Nivolumab monotherapy	Avelumab + axitinib	-	-	-	-	-
8	Sunitinib	Nivolumab monotherapy	Pembrolizumab + axitinib	-	-	-	-	-
9	Sunitinib	Axitinib	Nivolumab monotherapy	Pembrolizumab + axitinib	-	-	-	-
10	Sorafenib	Sunitinib	Everolimus	Axitinib	Nivolumab monotherapy	Pembrolizumab + axitinib	-	-
11	Nivolumab monotherapy	Sunitinib	Everolimus	Nivolumab monotherapy	Axitinib	Nivolumab + cabozantinib	-	-
12	Pazopanib	Pembrolizumab + axitinib	-	-	-	-	-	-
13	Ipilimumab + nivolumab	Nivolumab + cabozantinib	-	-	-	-	-	-
14	Ipilimumab + nivolumab	Avelumab + axitinib	Axitinib	-	-	-	-	-
15	Ipilimumab + nivolumab	Pembrolizumab + axitinib	-	-	-	-	-	-
16	Ipilimumab + nivolumab	Avelumab + axitinib	-	-	-	-	-	-
17	Ipilimumab + nivolumab	Pembrolizumab + axitinib	-	-	-	-	-	-

**Table 5:** Efficacy profile.

Characteristics	
Total, *n* (%)	17 (100)
Best response to IO+TKI combination therapy, *n* (%)	17 (100)
Complete response	0 (0.0)
Partial response	5 (29.4)
Stable disease	6 (35.3)
Progressive disease	6 (35.3)
Overall response rate, *n* (%)	
Yes	5 (29.4)
No	12 (70.6)
Disease control rate, *n* (%)	
Yes	11 (64.7)
No	6 (35.3)

IO+TKI: immuno-oncology plus tyrosine kinase inhibitor.

**Table 6:** AE profiles.

	Patients	
AE Profiles	Any Grade, *n* (%)	Grade ≥ 3, *n* (%)
Any event	16 (94.1)	7 (41.2)
Diarrhea	12 (70.6)	1 (5.9)
Hypertension	4 (23.5)	0
Fever	3 (17.6)	0
Hoarseness	3 (17.6)	0
Decreased appetite	2 (11.8)	0
Rash	2 (11.8)	0
Fatigue	2 (11.8)	1 (5.9)
Pneumonitis	2 (11.8)	1 (5.9)
Nausea	1 (5.9)	0
Alopecia areata	1 (5.9)	0
Hand–foot skin reaction	1 (5.9)	1 (5.9)
Diabetes mellitus	1 (5.9)	1 (5.9)
Thyroid dysfunction	1 (5.9)	0
Edema	1 (5.9)	1 (5.9)
Elevated serum creatine	1 (5.9)	0
Hyponatremia	1 (5.9)	1 (5.9)
Myasthenia gravis	1 (5.9)	1 (5.9)
Proteinuria	1 (5.9)	1 (5.9)

AE: adverse event.

**Table 7:** AEs of each patient.

Case Number	AEs	Grade	AEs	Grade	AEs	Grade	AEs	Grade
1	Diarrhea	1	Hand–foot skin reaction	3	Pneumonitis	3	-	-
2	Decreased appetite	1	Diarrhea	3	Hoarseness	1	-	-
3	Edema	3	Hypertension	1	-	-	-	-
4	Diabetes mellitus	4	Fever	1	Hyponatremia	3	-	-
5	Diarrhea	2	Fever	2	Hypertension	2	-	-
6	-	-	-	-	-	-	-	-
7	Fatigue	1	Hypertension	2	Nausea	2	-	-
8	Diarrhea	1	-	-	-	-	-	-
9	Diarrhea	1	-	-	-	-	-	-
10	Diarrhea	1	-	-	-	-	-	-
11	Diarrhea	1	Serum creatine elevation	2	Proteinuria	3	-	-
12	Diarrhea	1	Fever	1	Pneumonitis	1	Rash	2
13	Diarrhea	1	Fatigue	3	-	-	-	-
14	Diarrhea	1	Myasthenia gravis	3	Thyroid dysfunction	2	-	-
15	Alopecia areata	2	Hoarseness	1	Rash	2	-	-
16	Decreased appetite	2	Diarrhea	1	Hoarseness	1	-	-
17	Diarrhea	1	Hypertension	1	-	-	-	-

AE: adverse event.

**Table 8:** Reasons for discontinuing treatment.

Characteristics	
Total, *n* (%)	17 (100)
Patients who discontinued because of disease progression	8 (47.0)
Patients who discontinued because of AEs	3 (17.6)
Patients who experienced AEs attributable to IO therapy before IO+TKI combination therapy	6 (35.3)
Patients who discontinued because of AEs attributable to IO+TKI combination therapy among patients who experienced AEs attributable to pretreatment	2 (11.8)

IO+TKI: immuno-oncology plus tyrosine kinase inhibitor; AE: adverse event.

## Data Availability

We provided all data and methods in this manuscript.
